# The spatiotemporal evolution and influencing factors of hotel industry in the metropolitan area: An empirical study based on China

**DOI:** 10.1371/journal.pone.0231438

**Published:** 2020-05-19

**Authors:** Lei Li, Lin Lu, Yuchen Xu, Xiaolong Sun

**Affiliations:** 1 School of Geography and Tourism, Anhui Normal University, Wuhu, Anhui, China; 2 School of Tourism Management, Sun Yat-Sen University, Zhuhai, Guangdong, China; 3 School of International Tourism & Culture, Guizhou Normal University, Guiyang, Guizhou, China; Institute for Advanced Sustainability Studies, GERMANY

## Abstract

Through the online booking platform, 10,543 big data of spatial and temporal distribution of Beijing hotel industry has been obtained in this paper. Then, the methods of GIS and the geographical detector are used to study the spatiotemporal evolution process and the influencing factors of Beijing hotel industry during 2003–2018. The results are as follows: a. During the period of 2003–2018, the hotel industry in Beijing maintained a high growth rate and had three growth peaks in 2008, 2010 and 2014. Meanwhile, major historical events, such as the Olympic Games had a significant influence on the development of the hotel industry. b. Between 2003 and 2018, the hotel industry in Beijing gradually developed from the centripetal agglomeration to aggregation + diffusion, and also from the single center to the multi-center. Besides, various hotels presented two characteristics of city orientation and scenic orientation. c. The natural geographical environment had shaped the overall pattern and characteristics of the spatial distribution of the hotel industry in Beijing, and the socio-economic factors such as commercial activities, public facilities, tourism services and traffic conditions significantly influenced the location selection of the hotel industry. Therefore, the urban center is the ideal area for the spatial layout of the hotel industry. d. Geographical detector research showed that the factors, such as administrative organs, road network density, leisure and recreational facilities, and companies have strong explanatory power for hotel location selection, which is an important reference index for hotels to select the micro location. This paper is a beneficial supplement to the existing research and has certain guiding significance for the sustainable development of Beijing hotel industry.

## Introduction

Since the 1760 s, with the rise of the industrial revolution in Europe, cities have gradually replaced rural areas as the most concentrated areas of economic activities, which also gives rise to the concentration of various factors of production in urban areas. The rise of the city has promoted the prosperity of the business and service industries. Hence, in the early 19th century, modern hotels began to appear within the cities of Europe and the United States. By the early 20th century, it has formed a larger-scale hotel industry in New York, London, Madrid and other large cities in Europe and the United States [1,2]. After the end of the “Cold War” in the 1990 s, peace and development have become the mainstream of world development step by step. The world business and tourism activities have gradually emerged, and the hotel industry has entered an explosive high-speed growth stage under the background of economic globalization [3]. Especially in the Asia-Pacific region, a mass of large cities, such as Beijing, Hong Kong, Tokyo, and Seoul have emerged along with the rapid development of the social economy, and it has become one of the fastest growing and most concentrated areas in the global hotel industry.

The rapid development of the hotel industry has a key influence on promoting urban economic development, guiding urban industrial renewal and reshaping the internal structure of the city. Therefore, it has aroused the interest of scholars at home and abroad to carry out a lot of relevant research around the hotel industry. Among those research topics, the research on the evolution of the spatial-temporal pattern and influencing factors in the hotel industry has attracted the attention of geographers. In these studies, the method of description and statistical analysis are often adopted to analyze the evolution and location selection of hotels in early studies. For instance, the analysis of the interaction between the hotel and urban environment and transportation has pointed out that the hotel location should be given priority in the layout of the periphery of the city [4]. By analyzing the spatial growth process of the accommodation industry in the United States during 1963 to 1977, we have discovered that Central Florida, Las Vegas, Nevada, and Hawaii will be the main growth areas for the U.S. accommodation industry [5]. Through data analysis, the spatial distribution and evolution of different types of hotels in Toronto from 1950 to 1979 are described [6]. Moreover, with the deepening of the research, Ashworth first endeavored to explain the spatial distribution and evolution of the hotel industry from a theoretical perspective, and put forward the spatial distribution model of the hotel industry within the city [7]. Ashworth’s theoretical model has an important impact on subsequent research, and thus many scholars have verified the distribution patterns of hotels in different cities based on this theory [8–10]. At the same time, Egan has focused on the hotel’s micro-location options, believing that upscale hotels are more located in the center of the city, while midscale and ordinary hotels are apt to be located in the edge and the periphery of the city [11]. After the 21st century, with the spread of the global hotel industry, the focus of research has been gradually shifted to developing countries and cities where the hotel industry is developing faster [12]. Chinese scholars play a more pivotal role in this research, and they study the spatial layout and evolution of multinational hotels, star hotels, budget hotels and other types of hotels in mainland China [13–15], and pay attention to the impact of major events, such as the Olympic Games, the World Expo and the Canton Fair on the spatial pattern of the urban hotel industry [16,17]. They also discuss the spatial pattern and evolution of the hotel industry in large cities like Beijing, Shanghai, Guangzhou, etc. [10,16,18], and then they achieve abundant research results. Although the above-mentioned research concentrates on the spatial layout and the evolution process of the hotel industry, there are certain scholars trying to analyze the influencing factors of hotel location selection during the research process as well. For example, Ashworth has divided the hotel’s location selection into six types, which are the historical location, the railway location, the main road location, the small and medium-sized gathering location, and the large modern hotel location in the conversion area and the urban fringe location of motels and airports [7]. The follow-up scholars also study the effect on hotel location selection from the aspects of location features, the economic level, social culture, competition factors and the legal regulation [19]. Some Chinese scholars use case studies to conduct the empirical study on the factors influencing the spatial and temporal distribution of the hotel industry in Beijing, Wuhan, Guangzhou and other cities, which shows that traffic accessibility, commercial prosperity, and tourist attraction distribution are generally deemed to be the critical factors affecting the spatial layout of hotels [20–22]. Furthermore, some scholars also start from a single influencing factor, focusing on the impact of political factors, the resource acquisition, and road traffic on the spatial layout of the hotel industry within the city [23–25].

Through the above research, it can be observed that the study on the spatiotemporal evolution and influencing factors of the hotel industry has been a long time, and some research results have been obtained in the phenomenon description, factor exploration, regular deduction and the mechanism summary of the hotel industry’s spatial and temporal evolution. However, through the analysis of the above studies, it is not difficult to find that there is a key problem that the research data source is relatively single and the sample is normally small. Objectively speaking, thanks to the difficulty in obtaining long-term dynamic development data in the hotel industry, researchers often use sample data or type data obtained by statistical data, publications and data companies for replacement [5,6,10,16]. By reason of insufficient amount of data, it is hard for researchers to use these data to truly describe the spatiotemporal evolution of the hotel industry and interfere with the conclusions of the study. In recent years, the network platform provides and stores a large amount of data with the rapid development of internet technology, which provides vital data support for the study of spatial-temporal evolution of long-term and large-scale geographic things. On this basis, the study of the evolution of the spatial and temporal pattern in logistics, retail, cultural facilities and other industries using POI point data provided by electronic maps has been carried out [26–28]. Subsequently, some scholars have tried to use big data to study the spatiotemporal distribution of the hotel industry. They often obtain hotel data of Barcelona, Guangzhou and Wuhan through Airbnb, Google Earth and Baidu Map and other platforms to study the distribution pattern of the hotel industry in different regions [22,25,29]. Using big data to analyze the evolution process and the trend of spatial-temporal in the hotel industry is more comprehensive and accurate, which will become an important way and a trend to apply in related research. However, this research method is still in its infancy and the research results are still relatively few at present.

Based on the analysis and the summary of previous research, the author in this paper intends to treat Beijing as a case study to obtain the big data of the spatial and temporal distribution of the hotel industry through the hotel online booking platform, and then uses these big data to study the spatiotemporal evolution and influencing factors of the hotel industry in the metropolis and its surrounding areas. Beijing is a “first-tier city in the world” with a population of more than 20 million, and it is one of the most vital metropolises in the Asia-Pacific region and one of the earliest cities to develop hotels in modern China. After the reform and opening up, China’s economy has maintained a long-term high-speed growth, and the urbanization has been accelerating. As a national capital, Beijing also has achieved rapid development, and various industrial sectors there have been expanding rapidly. As an important part of business and tourism, the hotel industry has maintained an ideal development trend. As of 2018, Beijing has 198 multinational hotels, ranking first in the country and significantly ahead of other cities in China [13]. The hotel industry in Beijing has the typical characteristics of early development and the large scale. It has undergone a relatively complete evolution process and is influenced by multiple factors, such as economy, politics, transportation and tourism [10,20]. As the research object, Beijing has good representative and enlightenment for studying the spatiotemporal evolution process and influencing factors of the hotel industry in metropolitan areas. At the same time, this paper is expected to clarify the evolution law and the development trend of Beijing hotel industry, and provide some reference for promoting the sustainable development of the urban hotel industry.

## The overview of the research area, data and methods

### The overview of the research area

**Fig 1:**
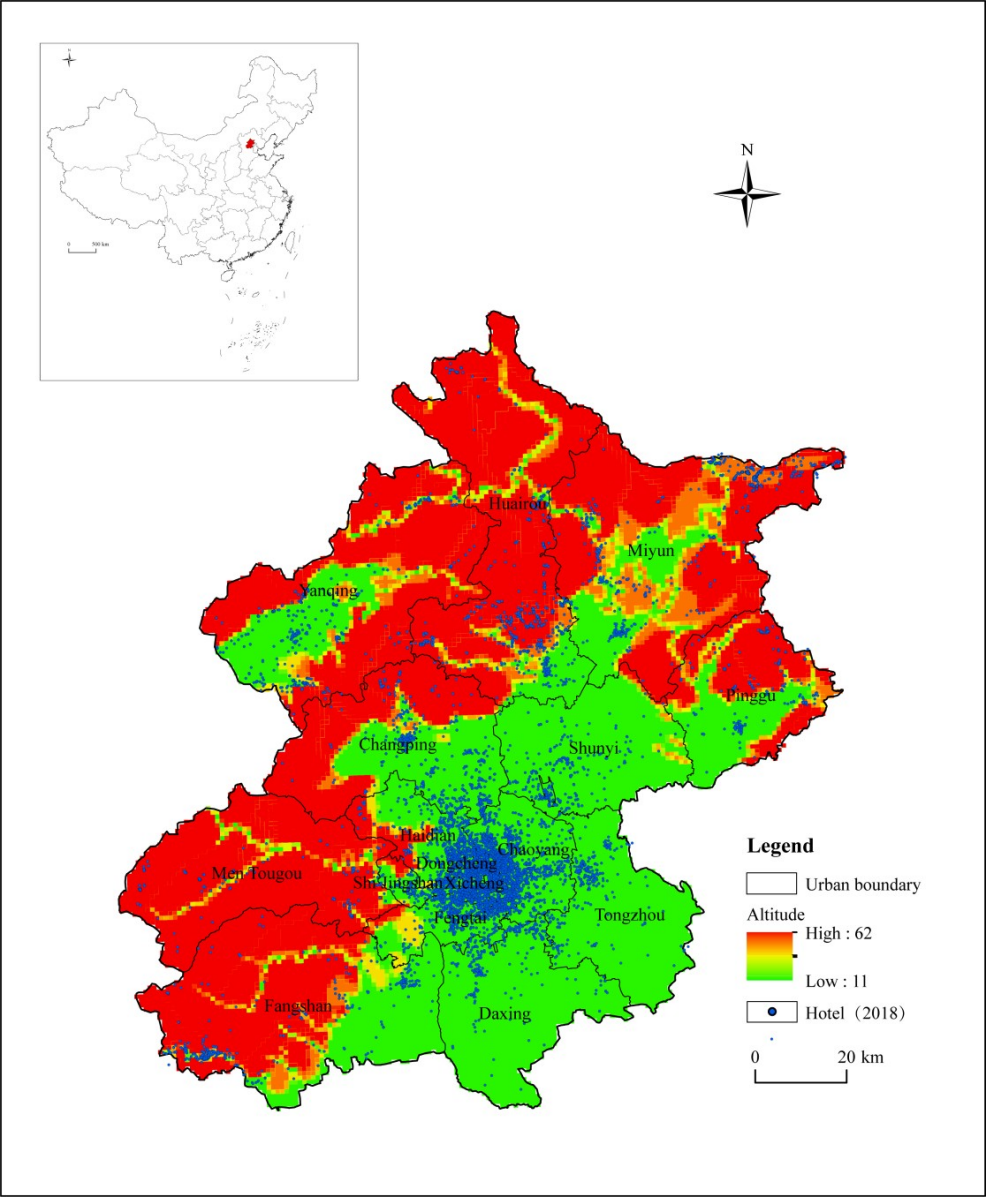
The overview of the study area and the study sample distribution. The color represents altitude, the darker the area, the higher the altitude. The blue dots represent all hotels in Beijing. The data comes from publicly available data on the Internet, which is organized by the authors themselves. The figure is drawn by Arc GIS.

Beijing is located in North China, between 115.7°~117.4°E and 39.4°~41.6°N, with a total area of about 16,410 km^2^ (Fig 1). The terrain of Beijing is high in the northwest and low in the southeast. Taihang Mountains and Yanshan Mountains are distributed in the west and north, while the southeast area is the North China Plain. The city’s altitude is between 8 and 2,303 m, and the average elevation is about 43.5 m. Beijing is the capital and national center of China, as well as the political, economic and cultural center of the country. The city contains 16 municipal districts, including 6 districts of the city center (Dongcheng, Xicheng, Chaoyang, Haidian, Fengtai, and Shijingshan), and 6 districts around the city (Shunyi, Tongzhou, Daxing, Fangshan, Mentougou, and Changping), and 4 districts in the periphery (Pinggu, Miyun, Huairou, and Yanqing). Beijing is one of the most developed regions in China. Its permanent population was 21.54 million and the GDP was RMB 3,032 billion at the end of 2018. Beijing has a complete urban transportation system, incorporating two international airports (Daxing International Airport, the Capital International Airport) and plentiful trunk railways and highways, and 16 subways and 303 subway stations have been built and operated. Besides, different types of economic formats, such as education, scientific research and business inside the city have been gathered as well. In addition to its outstanding political and economic functions, Beijing is still an important tourist city with more than 3,000 years of development history, and it has been chosen as the capital in six dynasties, such as Yuan, Ming and Qing. Beijing has rich tourism resources, covering 7 World Heritage sites like the Great Wall, the Forbidden City and the Summer Palace, and the 5A-level tourist attractions of the Olympic Park and Prince Kung’s Mansion. In 2018, the city received 310 million tourists, including 4.01 million inbound tourists. All of the good economic conditions, rich urban functions and huge tourism demand in Beijing have promoted the development of the city’s hotel industry. Currently, there are more than 10,000 hotels in operation in Beijing, which has formed a large hotel industry scale. With the construction of new cities, such as Xiong'an New District, Tongzhou and Yizhuang, the hotel industry in Beijing still has great development potential (Fig 1).

### Data

In this paper, the hotel space-time attribute data came from the hotel online booking network. Compared with POI data, the hotel online booking network provides detailed information, such as the price and opening time of each hotel, which is more in line with the needs of this study. Then, by comparing the online booking platforms of various hotels in China, we finally chose Qunar (https://www.qunar.com) and eLong (http://www.elong.com) as the data source website. These two websites are professional online hotel reservation platforms in China, they have a large number of Internet users and provide comprehensive hotel information. And, they allow researchers to get relevant web information, the collection method complied with the terms and conditions for the websites from which we have collected data, the similar research papers have been published [22].

In order to obtain research data, we choose Bazhuayu software (https://www.bazhuayu.com/) as a web information crawl tool, Bazhuayu is a professional crawler software that has been widely used in scientific research. By using the web crawler tool to capture the name, the address, opening time, the lowest price and other data of various hotels in Beijing, we grabbed 32,184 original data from Qunar and 11,789 original data from eLong. Combine the data obtained from the two websites, and used the Excel software for preliminary verification, by comparing hotel names and addresses, we delete the duplicate data. Afterwards, through manual screening, the closed, duplicated and missing hotel data were excluded, and finally 10,543 valid hotel data were obtained from the two websites.

Then, according to the name and the address of the hotel, the latitude and longitude query tool provided by Google Earth is utilized to obtain the spatial coordinate data. In the end, the method of random sampling is applied to select 3% (total 316) of the hotel latitude and longitude coordinate data in the Baidu Map coordinate query tool for verification, and the results show that the data match is 95.82%. The data of the matching degree is high, which can more accurately reflect the spatial distribution pattern of Beijing hotels (Fig 1). Through the above methods, the hotel space-time attribute data of Beijing required for this study were finally obtained.

The data used in the analysis of the factors affecting the distribution of the hotel industry were derived from *Beijing Regional Statistical Yearbook* (2018), the government department’s public data collation and Baidu POI data. All these data are public data, and the method of obtaining the data fully meets the relevant requirements, and the sources of each data are detailed in the influence factor analysis, which is not explained here.

### Methods

#### The standard deviational ellipse (SDE)

Standard differential ellipses are mainly used to describe the distribution direction and discreteness of geographical things, and its main parameters include the ellipse center, the long axis, the short axis and the azimuth. Among them, the ellipse center represents the center position of the thing; the long axis denotes the distribution direction of things; the short axis represents the distribution range of things. The shorter the short axis is, the more obvious the concentrated distribution trend of geographical things is. When the difference between the long axis and the short axis is larger (the flattening is larger), the directionality of the distribution of geographical things is more eminent [30–32]. The standard differential ellipse method is primarily used to study the spatial distribution traits and the evolution process of disparate types of hotels in Beijing, and the standard deviational ellipse tool of GIS software is used for calculation and analysis in this paper.

#### The nearest neighbor index (NNI)

The nearest neighbor index reflects the concentration of geographical things in space, and by comparing the nearest neighbor index of different things, we find it possible to describe and compare the concentration of different or identical things at different stages [33,34]. The nearest neighbor index is calculated as:

$$NNI=d\left(NN\right)/d\left(ran\right),\;d\left(ran\right)=1/\left(\sqrt{A/N}\right)$$ (1)

In Eq (1), *d* (*NN*) is the nearest neighbor distance; *d* (*ran*) is the expected average nearest neighbor distance; *N* is the number of samples; *A* is the area of the study area. When *d* (*NN*)>*d* (*ran*) and *NNI* <1, the study sample is concentrated; when *d* (*NN*)*<d* (*ran*) and *NNI* >1, the study sample is discretely distributed; when *d* (*NN*) = *d* (*ran*) and *NNI* = 1, the study sample is randomly distributed. The closer the *NNI* is to 1, the higher the probability of random distribution of the study sample is. The *Z* test is generally used to test the reliability of the research results.

In this paper, the nearest neighbor index was used to study the distribution and evolution of the hotel industry in Beijing. Because the nearest neighbor index is highly sensitive to the area in the study area, in order to ensure the reliability of the research results, the estimated area of Beijing was set to be the land area of 16,400 km^2^ when measuring the most adjacent point index of hotels at different times in Beijing.

#### The kernel density estimation (KDE)

Kernel density refers to the method of calculating the number or the core of geographical things within a certain range. The estimation point is deemed as the center; a certain distance is perceived as the search radius; then through the window movement operation the point or line density of each grid unit [31,34] is generated. Suppose *x*_*1*_, *x*_*2*_*… x*_*n*_ are the sample data in the density function *f*, and we estimate the value *f* (*x*) of *f* at a certain point *x* by the *Rosenblatt-Parzen* kernel function. The calculation method is:

$$f\left(x\right)=\frac{1}{nh}\sum_{i=1}^{n}k\left[\left(x-x_{i}\right)/h\right]$$ (2)

In Eq (2): *f* (*x*) represents the kernel density estimate of *f* at a certain point *x*; *k*[(*x*-*x*_*i*_)/*h*] represents the kernel function; (*x-xi*) denotes the distance at the valuation point *x* to *x*_*i*_; *h*>0 is the bandwidth.

In this paper, we used the kernel density estimation method to study the density and the evolution process of the spatial distribution of the hotel industry in Beijing. Since the kernel density of the point is rather sensitive to bandwidth selection, setting different bandwidths will bring about large differences in research results. Based on existing research [33–35], through trial and error, the bandwidth is finally set to 3 km in this study.

#### The geographical detector

The geographic detector is a research method to measure the spatial differentiation of geographical elements and analyze their causes, and it mainly includes four kinds of analytical methods, namely differentiation and factor detection, interaction detection, risk detection and ecological detection [36]. Compared with other methods of statistical analysis, the geographical detector can be used to analyze the influence of dependent variables on independent variables and their similarity in spatial distribution. The factor detector is chiefly used to detect the influencing factors of spatial analysis in Beijing hotel industry in this paper. The core idea is that if some influence factors and geographical changes have significant spatial consistency, this factor is decisive for the occurrence and development of geographical things [36]. Factor detection is calculated as:

$$q=1-\left[\left(\sum_{h=1}^{L}N_{h}\sigma_{h}^{2}\right)/\left(N\sigma^{2}\right)\right]=1-(SSW/SST)$$ (3)

In Eq (3), *h* represents the dependent variable *Y* or the strata of the factor *X; N and σ^2^* represent the total number of samples and variance; *N*_*h*_ *and* $\sigma_{h}^{2}$ denote the sample size and variance of the layer *h*; *SSW* and *SST* represent the with sum of squares and that of squares within the layer, respectively. The range of *q* is [0, 1]. The larger value of *q* represents the stronger explanatory power of the factor for the dependent variable *Y*. When *q* = 0, it indicates that the factor has no effect on the spatial distribution of *Y*. When *q* = 1, it signifies that the factor determines the spatial distribution of the dependent variable *Y*.

## Spatiotemporal distribution evolution of Beijing hotel industry

### The hotel classification in Beijing

Thanks to the great differences in the spatial distribution of different types of hotels, it is necessary to classify them. In previous studies, hotels were split into five-star, four-star, three-star or luxury, economical, etc. in line with the hotel’s own level [14,15], but this classification method ignored the hotel’s own attributes. Especially when the research sample was large, it was often difficult to accurately classify all hotels. Some researchers have suggested that the hotel industry resembles other commercial forms, and its selling price is the best embodiment of its own value, and so it can be classified by the hotel price [21,25]. Referring to the existing research results, we divided the hotel into three types: upscale, midscale and ordinary through the hotel price in this paper. The lowest price of upscale hotels should be greater than or equal to the average price of five-star and four-star hotels. The lowest price of midscale hotels should be greater than or equal to the average price of three-star hotels, but less than that of five-star and four-star hotels. The hotels with the lowest price being lower than the average price of three-star hotels were divided into ordinary hotels. The price of different star hotels was obtained by the hotel online booking platform using data crawling tools.

Through the methods, the final classification of upscale, midscale and ordinary hotels in Beijing is 1,128, 1,583 and 7,832, respectively, and the three types of hotels account for 10.70%, 15.01% and 74.29% of the total number of hotels in the city (Table 1). Overall, different levels of hotels in Beijing present a significant “pyramid” structure. Such a hierarchical structure can not merely meet the needs of different levels of consumers, but also reflect that the current hotel industry in Beijing is still serving the public consumers.

**Table 1:** The classification of hotels in Beijing.

Type	Sample	Sample size	Average price (RMB)	Total	Proportion
Upscale	five-star and four-star hotels	592	817 yuan or more	1128	10.70%
Mid-scale	three-star hotels	690	465–817 yuan	1583	15.01%
Ordinary	two-star and one-star hotels	—	less than 465 yuan	7832	74.29%

### Time evolution characteristics of Beijing hotel industry

**Fig 2:**
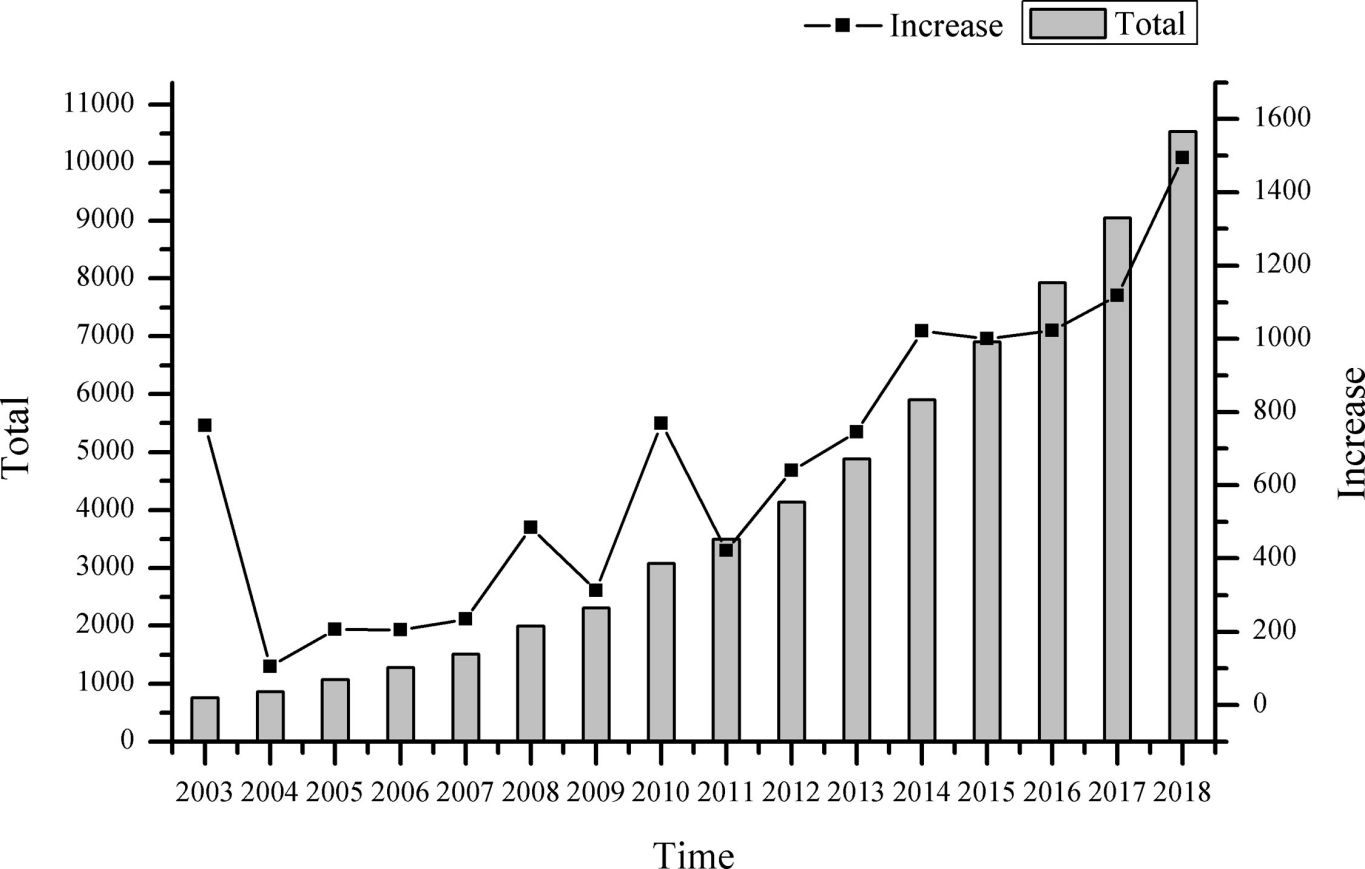
The evolution of hotel quantity in Beijing from 2003–2018. The histogram represents the total number of hotels in Beijing that year, and the line graph represents the number of new hotels in Beijing that year. The data comes from publicly available data on the Internet, the figure is drawn by Qrigin.

Among the 10,543 data, the hotel that started business earlier was Beijing Oriental Hotel in 1918. The time span of all hotels reached 100 years, and the hotel industry in Beijing has undergone a long evolution process. However, in the data obtained, the number of hotels in early Beijing was relatively small, and the operation to the present was very rare. Therefore, it has little significance to discuss the spatial distribution law alone. By drawing the annual variation of the number of hotels in Beijing (Fig 2), we found that the number of hotels in the city was relatively small before 2003. Besides, the annual growth is less than 100, and the annual change was smaller. Therefore, we take five years as an interval, and the spatial distribution and evolution of the hotel industry in Beijing on the four-time nodes in 2003/2008/2013/2018 are focused on in this paper.

In 2003, the total number of hotels in Beijing reached 763; the development of the hotel industry has begun to take shape; the number of hotels has maintained steady growth year by year; the overall development trend is ideal. In terms of interannual growth, the growth curve has three peaks, which are 2008, 2010 and 2014. Especially in 2008 and 2010, the growth rate reached more than twice that of the previous year, which was a period of rapid growth. In the past two years, the Olympic Games and the World Expo were held in Beijing and Shanghai, respectively. A mass of domestic and foreign tourists flocked to Beijing and other big cities to visit, and the surge in passenger flow has stimulated the rapid development of the hotel industry to a certain extent. From here, we can see that large-scale festivals and events have more significant impact on the hotel industry [16,17]. In 2014, the number of hotels in Beijing increased by more than 1,000 for the first time. In the five years from 2014 to 2018, the number of hotel growth in Beijing was more than 1,000 annually. Especially in the 2018, nearly 1,500 new hotels were added in Beijing, and the city’s hotel scale maintained a steady high-speed growth.

With the sustainable development of China’s economy in the future, the urbanization process will continue to accelerate, and the urban population will continue to grow. Therefore, the demand for the hotel industry will continually increase. At the same time, the hotel industry is also developing constantly, and new businesses, such as new homestays and shared accommodation are constantly emerging. In the future, the overall scale of the hotel industry in Beijing will continue to expand.

### Spatial evolution characteristics of Beijing hotel industry

#### The hotel industry center is always located in the main cities, distributed along the southwest-northeast direction in space

**Fig 3:**
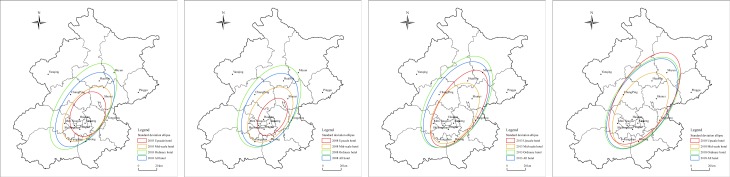
The standard deviation ellipse map of different types of hotels in Beijing 2003–2018. Different colors represent the spatial distribution of different types of hotel industry in Beijing. Among them, red represents the distribution of upscale hotels, orange represents the distribution of midscale hotels, green represents the distribution of ordinary hotels, and blue represents the distribution of all hotels. The figure is drawn by Arc GIS.

Comparing the standard deviational ellipse of different sorts of hotels in Beijing from 2003 to 2018 (Fig 3), we have found that the center of the city’s hotel industry is relatively clear, and various types of hotels have always concentrated in the urban center of the east and west. Over time, the center of the city’s hotel industry is slightly transferred to the northeast, indicating that the northeast direction of the city has become a new growth point for the hotel industry. The hotels in Beijing are mainly distributed along the southwest-northeast direction, with little change among different years. Although its distribution direction is restricted by the mountains and hills in the western and northern regions, it is also consistent with the distribution of cities and populations in the southeast region. Generally speaking, all kinds of hotels are distributed in a concentrated way in space. Especially in 2003–2008, the concentration of the hotel industry in the city has increased significantly because of the influence by Beijing Olympic Games. But after 2008, the trend of concentrated distribution has gradually weakened, and the hotel gradually expanded to the periphery of the city.

In terms of different types of hotels in Beijing, the center of the upscale hotels is most likely to be shifted to the northeast, and the ordinary and the midscale hotels are the least likely to change positions. For new upscale hotels, new towns or tourist areas away from the urban district are more possible to be chosen for the layout. Especially the north areas of Beijing, such as Miyun, Huairou have become the focus of the distribution of new upscale hotels. However, for midscale and ordinary hotels, there are higher requirements on the number of customers and strong dependence on the city center. Therefore, the layout of midscale and ordinary hotels is always around Dongcheng, Xicheng and other urban centers. At the same time, the spatial distribution direction of the upscale hotels changes most dramatically, and its distribution direction is the least obvious and mainly concentrated in the city before 2008. From 2008 to 2018, its southwest-northeast distribution trend significantly enhances, and the reason for this phenomenon may be the emergence of new upscale hotel clusters in the northeast region of Beijing. Meanwhile, the distribution of upscale hotels also spreads outward significantly when the center and direction shift. Before 2003, the city’s upscale hotels were mainly concentrated in the main urban areas of Dongcheng, Xicheng, Chaoyang and Haidian, while the trend of concentration further intensified in 2003–2008, but it continued to expand outward in 2008–2018. During the same period, owing to the impact of the city’s outward expansion, the distribution scope of midscale and ordinary hotels continued to expand, and the distribution direction is more evident.

#### The concentrated distribution of the hotel industry is obvious, and the spatial agglomeration of different types of hotels is strengthening

By calculating the nearest neighbor index of different types of hotels in Beijing from 2003 to 2018, we then described the distribution of hotels in Beijing (Table 2). The nearest neighbor indexes of all hotels at the four points 2003/2008/2013/2018 are 0.338, 0.312, 0.300, and 0.258, respectively, in which the Z values are all low and the P values are all less than 0.1. Through the significance level test, we find that the hotel industry in Beijing is generally in a state of agglomeration distribution at different time nodes, and the balance of hotel distribution is low. In the meanwhile, the nearest neighbor index of all hotels shows a slight downward trend in 2003–2018, indicating that the spatial concentration of the hotel industry in Beijing is gradually increasing.

**Table 2:** The nearest neighbor index of different types of hotels in Beijing from 2003–2018.

Time	Index	All	Upscale	Midscale	Ordinary
2003	Observed mean distance (m)	918.23	3952.12	1535.48	1383.35
	Expected mean distance (m)	2718.17	5234.48	3793.12	3612.51
	Nearest neighbor ratio	0.338	0.755	0.405	0.383
	*z* - score	-34.992	-3.569	-18.882	-24.479
	*p* - value	0.000	0.000	0.000	0.000
2008	Observed mean distance (m)	543.74	1962.15	1183.28	730.65
	Expected mean distance (m)	1745.51	3871.14	2970.85	2213.36
	Nearest neighbor ratio	0.312	0.507	0.398	0.330
	*z* - score	-58.860	-11.477	-28.360	-45.164
	*p* - value	0.000	0.000	0.000	0.000
2013	Observed mean distance (m)	345.18	1311.03	896.26	410.10
	Expected mean distance (m)	1149.03	3135.65	2440.10	1356.07
	Nearest neighbor ratio	0.300	0.418	0.367	0.302
	*z* - score	-93.542	-24.717	-36.493	-78.760
	*p* - value	0.000	0.000	0.000	0.000
2018	Observed mean distance (m)	202.59	834.40	701.19	246.60
	Expected mean distance (m)	784.38	2290.46	1879.55	910.00
	Nearest neighbor ratio	0.258	0.364	0.373	0.271
	*z* - score	-145.698	-40.845	-47.720	-123.423
	*p* - value	0.000	0.000	0.000	0.000

Observed mean distance (OMD), average distance of hotels randomly distributed in Beijing; Expected mean distance (EMD), actual average distance of hotel distribution in Beijing; Nearest neighbor ratio (NNI): *NNI* = *OMD*/*EMD*, The closer the *NNI* is to 1, the higher the probability of random distribution of the study sample is. *Z* and *p* are generally used to test the reliability of the research results.

In terms of various hotels in Beijing, the concentration distribution of ordinary hotels is the most prominent at different stages, followed by the midscale type. And the nearest neighbor index of the two types of hotels shows a slight downward trend, while the distribution of agglomeration is enhanced. Although the upscale hotels also showed an agglomeration distribution trend, its nearest neighbor index before 2008 is significantly higher than that of the midscale and ordinary types, and its aggregation intensity is the weakest among the aggregation intensity of the three types of hotels. By 2018, the nearest neighbor index of the upscale hotels has dropped from 0.755 to 0.364, and its spatial agglomeration has significantly enhanced. However, this is different from the research conclusion of the standard deviational ellipse, and the reasons for this need to be further explored through kernel density estimation.

#### The concentration core of the hotel industry continues to strengthen, and the hotels in the peripheral areas also gain certain growth

**Fig 4:**
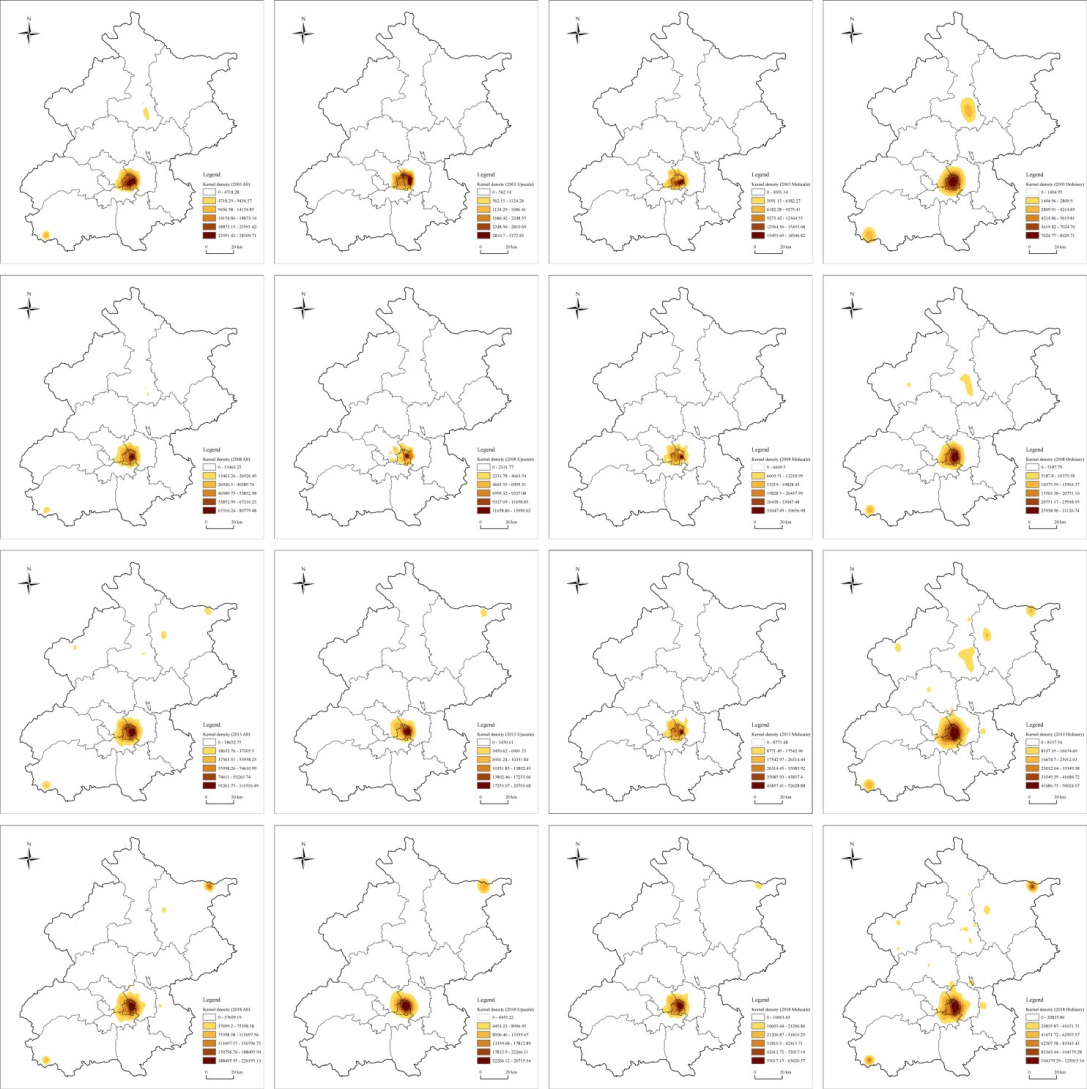
The kernel density estimation map of different types of hotels in Beijing 2003–2018. The color represents the nuclear density of the spatial distribution of different types of hotels in Beijing. The darker the color, the greater the density of the hotels in the area. The map is calculated and drawn by Arc GIS according to formula (2).

Comparing the kernel density distribution of different types of hotels in Beijing from 2003 to 2018 (Fig 4), we can observe that the concentration distribution trend of hotels in the whole city is obvious, and the core is concentrated in the regions of Dongcheng and Xicheng, as well as the surrounding areas of Haidian, Chaoyang and Fengtai districts. Moreover, the kernel density of the agglomerated core increased significantly in 2003–2018. The maximum kernel density in 2003 is about 28,309.71, and it increased to 226,195.13 in 2018. It increased by about 10 times in 15 years, showing that although the concentration of the core of the cluster does not change much, the number of its internal hotels has increased significantly in fact and the core of aggregation is constantly strengthening. Furthermore, although the peripheral hotels in the urban areas have also achieved certain growth, the spatial distribution of peripheral hotels was relatively scattered (Fig 1). In order to maintain the comparability of the data, the bandwidth of the kernel density estimation is always set to 3 km in this paper, and so the kernel density of the peripheral hotels is always small. In addition to the agglomeration core, there are two sub-cores of Beijing hotel industry in 2003, which are located in Huairou and the south of Fangshan (Shidu Scenic Area). By 2018, a number of sub-cores appeared in the city's hotel industry, and they are located in Tongzhou, the southern part of Fangshan (Shidu Scenic Area), the northern part of Miyun (Gubei Water Town Scenic Area) and the western part of Miyun (Miyun Reservoir Scenic Area). As the administrative sub-center of Beijing, Tongzhou has relatively rapid urban construction and development, and has great potential for future development, and thus it has turned into a key area for the layout of the current hotel industry. As famous scenic spots around Beijing, Gubei Water Town, Shidu Scenic Area and Miyun Reservoir are important destinations for tourists from other places and places for weekend leisure for local citizens. There are 389, 1133 and 166 hotels in the surrounding 5 km area. Especially for Gubei Water Town built in 2010, it has become one of the fastest growing areas in Beijing. Based on this, it can be seen that Beijing hotels mainly include urban-oriented and scenic-oriented types, and the future new city areas and key scenic spots will also become the main areas for the growth of the hotel industry.

As far as different types of hotels in Beijing are concerned, the concentration core of upscale hotels is the clearest. Although it moves slightly to the northeast in 2003–2018, the concentration core has been distributed in the main urban area between Dongcheng and Chaoyang. However, among the three types of hotels, the upscale hotels have the smallest kernel density and the slowest growth, indicating that the distribution of upscale hotels within the concentration center is scattered. The reason for this phenomenon may be that there are high requirements on the customer base for upscale hotels and fierce competition among different hotels. After 2013, Gubei Water Town has gradually become the sub-center of upscale hotel gathering. By 2018, the density of the Gubei Water Town has been continuously improved, and it has become a significant gathering area for emerging upscale hotels in Beijing, which also explains the above analysis results of the standard deviational ellipse and the nearest neighbor index. In fact, the upscale hotels in Beijing are still concentratedly distributed, and the distribution center and direction are moving greatly to the northeast. The fundamental reason is the huge growth of the upscale hotels in Gubei Water Town and its surrounding areas.

The kernel density of the midscale hotels is always high, and its spatial agglomeration and scale effect are the strongest. The midscale hotels have a relatively stable concentration center, and they are always concentrated in the central areas of Dongcheng, Xicheng, Chaoyang, and Haidian. Affected by the population, traffic and so on, the midscale hotels show strong dependence on the city center. In addition, with the largest base, the largest increase in 15 years, and the fastest growth in density, the ordinary hotels account for 74.29% of the total number of hotels. Although the ordinary hotels are always located in the core of aggregation in the city center, the randomness of distribution is obviously stronger than that of upscale and midscale hotels. In 2018, a number of concentration sub-cores have been formed in the surrounding urban areas of Gubei Water Town and Shidu Scenic Area.

### Analysis of the influence factors of the spatiotemporal distribution in Beijing hotel industry

The spatial distribution of geographical things is influenced by both the natural geographical environment and the social economic environment [34], and the distribution rules of the same geographical things in different spaces and different geographical things in the same space are disparate. In order to explore the influencing factors of the spatial distribution of the hotel industry in Beijing, the number of different types of hotels in the 16 municipal districts of Beijing is taken as the dependent variable, and qualitative analysis and the geographical detector are used to study them based on both natural geographical factors and socio-economic factors in this paper. Due to the adjustment of administrative divisions and the significant differences of influencing factors in different historical periods (such as the Olympic Games), this study simply analyzes the influencing factors of the location distribution of the hotel industry in 2018.

### Natural geographical factors

The natural geographical environment has an important impact on the spatial distribution of the hotel industry. The plain areas with flat terrain, the low slope, the abundant water source and suitable environment are more likely to become the hotel industry cluster area. The overall terrain of Beijing is high in the northwest and low in the southeast. Miyun, Huairou, and Yanqing in the north and Mentougou and Fangshan in the west are distributed in the Yanshan and Taihang Mountains, and the average elevation of these areas is between 1000–1500 m. The terrain is more complex, but is also a crucial ecological barrier in Beijing. So, it is not suitable for large-scale human economic activities, and the hotel is sparsely distributed (Fig 1). Taking Mentougou District as an example, its area is about 1,451 km^2^, accounting for 8.84% of the total area of Beijing and ranking 5th in the city. But the total number of hotels in Mentougou is merely 237, accounting for 2.25% of the city’s total and ranking 13th. The southeast region in Beijing is dominated by plains and has a flat terrain, which is the main concentration area of cities and populations. There are the traditional “six districts in Beijing”, such as Dongcheng and Xicheng, as well as future new cities, such as Tongzhou, Shunyi and Yizhuang. The scale of development and the potential for development in those areas are both large, which makes these areas densely distributed areas for hotels in Beijing (Fig 1). At the same time, because of the special geographical environment of Beijing, the city’s hotel industry center is distributed in the southeastern urban areas, and the hotel industry is generally distributed in the northwest-southeast direction (Fig 3).

### Social and economic factors

Social and economic conditions are pivotal factors influencing the choice of space in the hotel industry. Researchers at home and abroad have conducted relevant research from the regional economy [11,19,21], traffic conditions [4,7,20,21,25], business conditions [18,37,22], public services [19,22,37], tourism situations [20,29] and other aspects. Based on the reference of existing research results, we take into account the scale, the characteristics and the availability of the research data. Finally, the dependent variables of 5 indicators and 19 factors (X1,X2… X19) affecting the spatial distribution of the hotel industry in this paper are determined, among which the dependent variables are Y1 (the total number of hotels in a certain district), Y2 (the number of upscale hotels in a certain district), Y3 (the number of midscale hotels in a certain district) and Y4 (the number of ordinary hotels in a certain district). It should be stated that special historical events, such as the Olympic Games and the World Expo exert an important influence on the location selection of the hotel industry [16,17], but the cross-sectional data of 2018 are mainly used for research in this paper, and so these major historical events are not considered in this study (Table 3).

**Table 3:** The factors of hotels’ spatial distribution in Beijing.

Index	Factor	Indicator meaning (2018)	Sources
Regional economy	X1: Per capita GDP	Average gross national product of the permanent population	①
	X2: Per capita disposable income	Everyone's discretionary income	①
	X3: Permanent population density	Resident population density	①
	X4: Land price	Average land transaction price	②
Traffic condition	X5: Train passenger capacity	Weighted scores of train passenger capacity (using the assignment method, according to the number of trains arriving at the station): G/C/D-train = 10, Z/T-train = 4, K/other-train = 2	②
	X6: Subway station	Number of completed subway stations	②
	X7: Per capita car ownership	Number of cars owned by everyone	①
	X8: Road density	Navigating road density	①
Business condition	X9: Company	Total number of companies	③
	X10: Restaurant	Total number of restaurants	③
	X11: Leisure and recreational facilities	Total number of leisure and recreational facilities	③
	X12: Convenient service facilities	Total number of convenient service facilities	③
Public service	X13: Administrative agency	Total number of administrative agencies	③
	X14: Bank and Insurance	Total number of banks and Insurance	①
	X15: Hospital	Total number of hospitals	①
	X16: University and college	Total number of universities and colleges	②
Tourism situation	X17: Tourism resource	Weighted scores of tourism resource (using the assignment method based on the resource level and only counting A-level scenic spots): 5A = 10, 4A = 8, 3A = 6, 2A = 4, 1A = 2	②
	X18: Travel agency	Total number of travel agency	②
	X19: Inbound tourists	Total number of inbound tourists	①

The research data sources are: ① *Beijing Regional Statistical Yearbook* (2018), ② government department public data collation, ③ Baidu POI data, and ④ train passenger capacity and tourism resource endowment reference related research in accordance with the assignment method [22,38]. All the data were finally input into the GIS software. Then, according to the Jenks natural fracture classification method, it was divided into the 1–5 sequence data with the smallest internal difference and the largest difference [28,36]. Finally, the influence of various factors on the spatial distribution of hotels is analyzed via using the geographic detector software (Table 3).

**Fig 5:**
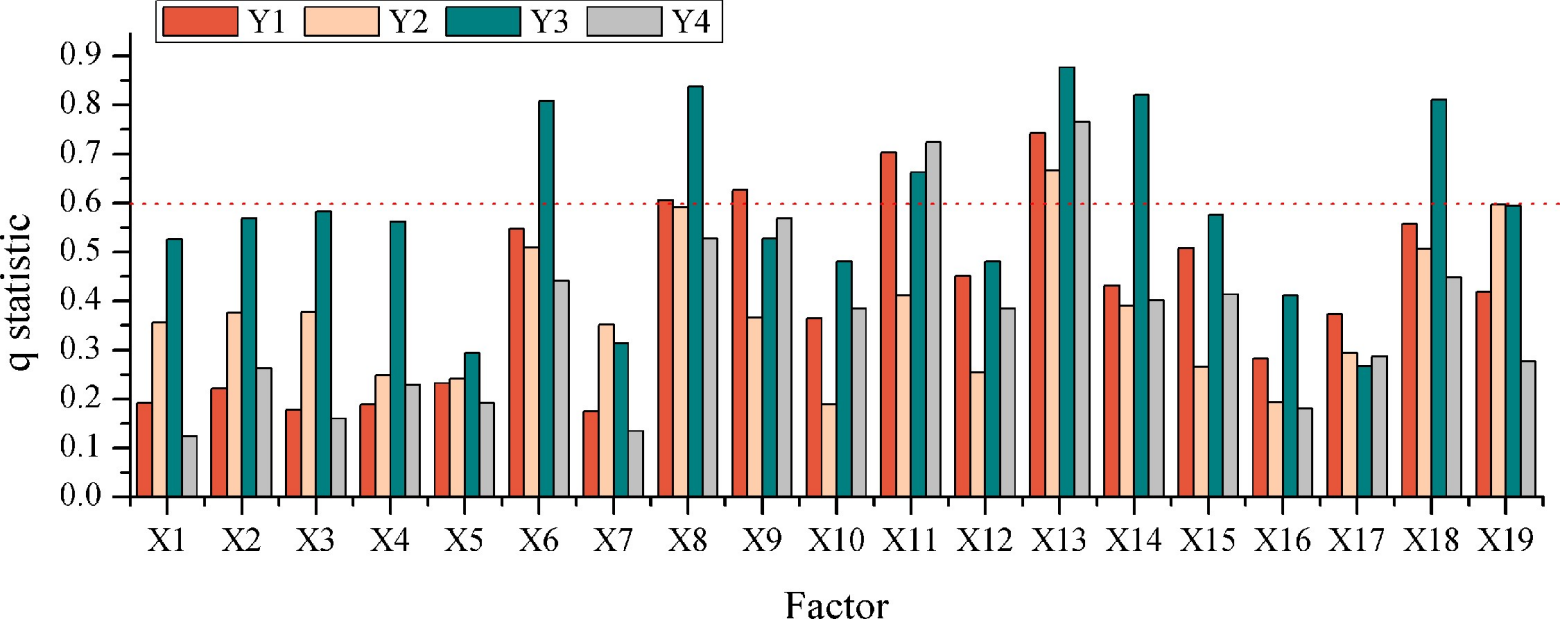
Analysis results of the geographical detector (q). The X-axis represents 19 factors that influence the spatial distribution of Beijing's hotel industry, and the Y-axis represents the degree of influence of different factors on the spatial distribution of Beijing's hotel industry. The data is calculated by formula (3), the figure is drawn by Qrigin.

The results of geographical detector analysis show that the factors affecting the spatial distribution of different types of hotels are quite unlike (Fig 5). According to the analysis results of each factor in the 5 indicators, the impact of the indicators on the spatial distribution of the hotel industry in Beijing is calculated, and then the identification of q ≥ 0.60 (last rounding) is a significant factor in the spatial distribution of the hotel industry (Table 4). The results of the study reflect that:

**Table 4:** The average value of hotel industry factors and the significant impact factors in Beijing.

Index	Y1	Y2	Y3	Y4
Regional economy	0.1946	0.3397	0.5598	0.1938
Traffic condition	0.3904	0.4232	0.5632	0.2901
Business condition	0.5360	0.3051	0.5376	0.5149
Public service	0.4910	0.3787	0.6708	0.4403
Tourism situation	0.4495	0.4653	0.5570	0.3372
Significant influence factor	X13, X11, X9, X8	X13, X19	X13, X8, X14, X18, X6, X11	X13, X11

Y1, the total number of hotels in a certain district; Y2, the number of upscale hotels in a certain district; Y3, the number of midscale hotels in a certain district; Y4, the number of ordinary hotels in a certain district.

The factors affecting the spatial distribution of all hotels in Beijing are listed as follows: the business condition (0.5360) > public service (0.4910) > the tourism situation (0.4495) > the traffic condition (0.3904) > the regional economy (0.1946). Among them, X13 (the administrative agency), X11 (leisure and recreational facilities), X9 (companies) and X8 (road density) are significant influence factors. The area with developed business and complete public facilities has abundant business and official floating populations, and it also has strong consumption capacity and accommodation demand, which greatly promotes the development of the hotel industry in the surrounding areas. Due to this reason, the hotel industry in Beijing has been spatially distributed with administrative agencies and companies, and it always forms an obvious agglomeration core in the main urban areas, such as Dongcheng, Xicheng, Chaoyang and Haidian. Furthermore, tourism and traffic facilities also have a prominent impact on the location selection of the hotel industry in Beijing, especially from the micro perspective. There are numerous hotels distributed around the main scenic spots, such as Nanluoguxiang and Gubei Water Town, and the important transportation hubs, such as Beijing South Railway Station and Beijing West Railway Station. They also formed an industry agglomeration area. But when the research unit is enlarged to a district, its influence will decline. Relatively, regional economic conditions have the least significant impact on the spatial distribution of the hotel industry in Beijing, and the reason for this phenomenon may lies in that the hotel mainly serves the external population related to business, government, and tourism, instead of local residents who have less demand for hotels.

In the case of different types of hotels, the tourism situation and traffic conditions have the most significant impact on the spatial distribution of the upscale hotels. X13 (the administrative agency) and X19 (inbound tourists) best explain the factors of the spatial distribution of upscale hotels, which is the significant influencing factor. It is well known that the hotel is a key part of the tourism industry, while the development of the tourism industry provides the hotel with plenty of customers having high consumer demand, and the two rely on each other and complement each other. Beijing tourism resources are primarily concentrated in the central and northern regions, of which Dongcheng, Xicheng, Chaoyang and other urban areas in the central have developed earlier and have a larger scale, and so there have always been a concentrated distribution area of upscale hotels. In recent years, with the development of tourism and the improvement of traffic conditions in the northern Miyun, Huairou and other areas, it is becoming a new growth center for upscale hotels (Fig 3). Besides, because the area with good traffic location conditions has a significant passenger flow advantage, it is also an important choice in the layout of upscale hotels. Despite the obvious edges in the main city, there are significant “market thresholds” for upscale hotels, and it brings about the fierce competition between the hotels. In reality, the density growth of upscale hotels inside the main city has slowed down after 2013 (Fig 4), while Tongzhou, Shunyi and other peripheral new cities, Daxing International Airport, Beijing South Station and other transportation hubs, Gubei Water Town, the Yanqi Lake and other key scenic spots will become important choices for the space layout of upscale hotels in Beijing in the future.

Moreover, the factors affecting the spatial distribution of midscale hotels are as follows: public service (0.6708) > the traffic condition (0.5632) > the regional economy (0.5598) > the tourism situation (0.5570) > the business condition (0.5370). The significant impact factors include X13 (the administrative agency), X8 (road density), X14 (the bank and insurance), X18 (the travel agency), X6 (the subway station) and X11 (leisure and recreational facilities). Different from other types of hotels, the five research indicators have a significant impact on the spatial distribution of midscale hotels in Beijing, and the average explanatory power of factors has reached more than 0.53. In the actual research process, the areas with perfect public services, good traffic conditions, the developed regional economy, complete tourism situations and frequent business activities are apt to concentrate in the city center, which has given rise to the obvious distribution of the midscale hotels in Beijing. Thus, the cluster center of the midscale hotels in Beijing has been continuously strengthened and has been stably concentrated in the central areas of Dongcheng, Xicheng, Chaoyang and Haidian (Fig 3). Based on this, it can be seen that midscale hotels have strong urban orientation, which is inclined be accompanied by the administrative agencies and bank insurance institutions in the city. Because of the obvious location advantage of the city center, its function is difficult to be moved out in the short term. Although the growth rate may slow down, the agglomeration core of mid-level hotels in the future will still be concentrated in the main urban area, but the agglomeration sub-core may appear in new city areas, such as Tongzhou and Yizhuang.

The more significant influence on the spatial distribution of ordinary hotels is the degree of the business condition and public service, while the traffic condition and the regional economy have a low impact on them, and the significant factors with higher explanatory power are X13 (the administrative agency) and X11 (leisure and recreational facilities). Thanks to the large number of ordinary hotels, it is in accord with the overall distribution of all hotels, and it tends to be distributed in urban centers with developed commercial and public service facilities. Meanwhile, ordinary hotels have the fastest growth rate, and the density is far higher than that of other types of hotels. Unlike upscale and midscale hotels, ordinary hotels are not only numerous, but also richer in types. It includes economic accommodation for business and official business, as well as many farmhouses and country hotels service for tourists and local citizens. In terms of spatial distribution, the ordinary hotels are not sensitive to traffic and regional economic conditions, and their distribution scope is not concentrated in a certain area, but more dispersed in spatial distribution. Therefore, several secondary agglomeration cores are formed in the centers of districts and the key scenic spots outside the city (Fig 4). Because of the large base, the low cost and the short life cycle of the ordinary hotel, the city will still be the main growth area in the future, but the peripheral urban area, the new city center, tourist attractions and other areas may become the new growth point of ordinary hotels in Beijing.

Among the 19 factors affecting the spatial distribution of the hotel industry in Beijing, the strong explanatory powers include: X13 (the administrative agency), X8 (road density), X11 (leisure and recreational facilities), X18 (the travel agency), X6 (the subway station), X9 (companies) and X14 (the bank and insurance). Among them, X13 (the administrative agency) has a significant impact on the number of different sorts of hotels. As the capital of China, Beijing has a strong national administrative function. Every year, there are a lot of official passengers traveling to Beijing, and so the influence of the administrative organs will be fully considered and hotels will tend to be laid out in the surrounding areas of the administrative agencies in the selection process of the city’s hotels. Beyond that, other more significant factors mainly include the urban internal traffic condition and the regional economy, and the areas along traffic trunk lines and metro lines are critical choices for the layout of the hotel industry. Nevertheless, companies, the bank and insurance, and leisure facilities are more inclined to accompany the distribution of the hotel industry, in order to share each other’s customers to enhance economy benefits of the scale.

The weaker explanatory factors for the spatial distribution of hotels in Beijing include: X3 (permanent population density), X4 (the land price), X17 (the tourism resource), X1 (the per capita GDP), X16 (the university and college), X7 (per capita car ownership) and X5 (train passenger capacity). Among them, X3 (permanent population density), X4 (the land price), X1 (the per capita GDP), and X7 (per capita car ownership) can be classified into the regional economy, for these factors mainly reflect the local economy, while the hotel industry is mainly in the service of floating population, and so the factor explanatory power is generally weak. This differs from previous studies [20,21], which may result from different research units. The spatial distribution of X17 (the tourism resource), X16 (the university and college), and X5 (train passenger capacity) is extremely uneven, with significant geographical concentration. For example, the institutions of higher learning are mainly distributed in Haidian District in Beijing, and the railway station is mainly concentrated in Fengtai, or in other areas with the very low score, and thus the explanatory power of the factors is weak during the analysis. If we start from a smaller scale, the explanatory power of factors may vary significantly.

## Discussion

As the capital city of developing countries, Beijing hotel industry has reached a peak of development after 2000, which is significantly later than the time of developed countries and regions in Europe and America, such as New York and Toronto [5,6]. However, the development of the hotel industry in Beijing is relatively fast, and it has formed a large industrial scale in the short run. There are significant differences in interannual variations, and there are “peak value” and “valley value” of growth (Fig 2). Furthermore, the discrepancies between the interannual changes have confirmed that the key historical events, such as the Olympic Games, have a major impact on the development of the hotel industry [16,39], and can often tremendously promote the development of the urban hotel industry in the short term [16,40].

The spatial evolution process of the hotel industry in Beijing has a certain similarity with some cities, such as Toronto, Istanbul, Guangzhou, and Wuhan [6,18,22]. In the early stage of development, the hotel industry was mainly concentrated in the urban center area. With the influencing factor of the saturated inner market in the city, increased competition and urban expansion, although the focus of development is still concentrated in the city, the hotel industry has gradually begun to expand to the peripheral areas [22,37]. Several secondary agglomeration cores have been formed in the satellite city, tourist attractions and other urban peripheral areas. The whole hotel industry presents a development and evolution situation of “agglomeration” to “aggregation + diffusion”, from the “single center” to the “multi-center” [18,41,42]. Different from previous studies which pay more attention to the evolution process of the same type of the hotel or the distribution pattern of different types of hotels [10,15], the hotel in Beijing is divided into three types, namely upscale, midscale and ordinary types, according to the unit price of hotel rooms, and then its evolution process is studied separately in this paper. It is found that the spatial distribution characteristics and the evolution process of different types of hotels have certain differences. The upscale hotels tend to be distributed in urban centers, and key scenic spots, midscale hotels are concentrated in the city center. But the ordinary hotels are randomly distributed, with its center being concentrated in the city center, but easy to form a number of secondary cluster cores on the periphery (Fig 4). Therefore, various hotels in Beijing show two traits of urban orientation and scenic orientation [29,41].

The spatial distribution of the hotel industry is influenced by the combined influence of natural geographical, environmental and socio-economic factors [37,41]. Based on the empirical analysis of the hotel industry in Beijing in this paper, it is reflected that the natural geographical environment has a significant impact on the overall pattern of the hotel industry. It also verified that the business condition, the public service, the tourism situation and the traffic condition greatly impact the spatial distribution of the hotel industry [7,18–22,37,43]. However, unlike previous studies, the impact of the regional economy on the location choice of the hotel industry is not significant [11,19,21]. At the urban scale, the regional economy mainly reflects the economic status and consumption power of local residents, while the hotel mainly serves the floating population, and the accommodation needs of local residents are relatively low, and so there is no significant correlation between the two (Table 4). In terms of the influence factors of the spatial distribution of different types of hotels, the upscale hotels are most affected by the tourism situation and traffic conditions, and the midscale hotels are more significantly affected by public service, the traffic condition, the regional economy, the tourism situation and the business condition; ordinary hotels are affected by the business condition and public service, which generates differences in spatial distribution (Table 4 and Fig 4).

Through the analysis of 19 influencing factors affecting the spatial distribution of the hotel industry, this paper verified that road density, leisure and recreational facilities, the travel agency, the subway station, the company, the bank and insurance significantly influence the spatial distribution and location selection of the hotel industry [20,21], which has a significant agglomeration distribution in space. However, different from the previous studies [20,21], this paper found that the administrative agency has strong explanatory power for the spatial distribution of all types of hotels, which reflects that Beijing as a capital has a strong administrative service function, and this characteristic may be common in the capital city. Moreover, the factors that have weaker interpretation of the hotel's spatial distribution factors include the permanent population density, the land price, and the per capita GDP, which are regional economic conditions. They also cover the extremely uneven spatial distribution factors, such as the tourism resource, the university and college, and train passenger capacity. Although these factors may be critical factors in the selection of hotel micro-locations [20,21], they have no prominent impact on the number of hotels in a district. In particular, in this paper land prices are believed to have a weak influence on the spatial distribution of hotels, which is significantly different from previous studies [11,20,22]. In fact, the actual study found that many hotels currently take the form of leasing operations, and so they don’t need to invest in construction. Consequently, the impact of land prices on their location selection is unobvious.

Compared with conventional research, the application of big data to study the spatiotemporal distribution and influencing factors of the hotel industry has the following three advantages. First, the data acquisition path is optimized, which greatly supplements the data volume of the research sample. Traditional research is limited by data acquisition channels, and merely a small amount of sample data can be used for related research in general [10,20]. But big data technology provides an important way to obtain long-term hotel evolution data, while the obtained data are used to establish the dynamic evolution database of the hotel industry in Beijing. It will provide important support to observe the spatiotemporal evolution of Beijing hotel industry for the future [22,44]. Second, it has enhanced the research on the influencing factors of hotel spatial distribution, especially the data in connection with other geographical things, such as the business condition. Traditional research is mostly limited by the availability of data, making it often difficult to obtain effective research data in the selection of influencing factors [20] or making it forced to use qualitative research methods for analysis [18,37]. When studying the influencing factors of spatial distribution of Beijing hotel industry, we obtained about 200,000 pieces of spatial attribute data of companies, restaurants, leisure and recreational facilities and other related geographical things in Beijing through big data technology, and further analyzed the interaction between different geographical things in this paper. Third, the use of big data further enhances the authenticity and reliability of the research conclusions. Because the sample size of this study is much larger than that of previous studies [10,20,21], it not only reflects the spatial distribution and the evolution process of the hotel industry more realistically, but also avoids the interference caused by qualitative analysis and the artificial assignment of data in previous studies in the analysis of influencing factors. Besides, it enhances the value of the research conclusions.

Finally, through the analysis of big data and the research on the spatial distribution characteristics and influencing factors of Beijing hotels, we can predict the development of different types of hotels in Beijing in the future and provide guidance for the sustainable development of the city’s hotel industry. Upscale hotels are largely affected by the tourism situation and the traffic condition, and tend to be located in urban centers, transportation hubs, key scenic spots, and peripheral new cities. Therefore, Tongzhou, Shunyi and other peripheral new cities, Daxing International Airport, Beijing South Station and other transportation hubs, Gubeishui Water Town, Yanqi Lake and other key scenic spots will become a significant choice for the space layout of upscale hotels in Beijing in the future. Midscale hotels have high requirements for all facilities and have strong urban orientation. They will continue to be concentrated in the main urban centers in years to come, but new growth centers may appear in new city areas which are developing rapidly, such as Tongzhou and Yizhuang. Ordinary hotels are not sensitive to the traffic location and the regional economy. Although the city center is still the prime growth area of the future, the peripheral urban area, the new city center, tourist attractions and other areas may become the new growth point of ordinary hotels in Beijing.

## Conclusions

Through the study of the spatial and temporal evolution and influencing factors of the hotel industry in Beijing during 2003–2018, the following conclusions can be drawn.

During the period of 2003–2018, the hotel industry maintained a high growth rate in Beijing, and had three growth peaks in 2008, 2010 and 2014 successively. Meanwhile, major historical events, such as the Olympic Games and the World Expo, have a significant impact on the development of the hotel industry [16,17]. Currently, the hotel industry in the city has formed three basic types, namely upscale, midscale and ordinary types, which are distributed in a “pyramid” shape. At present, the hotel industry is still to serve the public consumers.

Between 2003 and 2018, the city’s hotel industry coexisted in agglomeration and diffusion, and gradually evolved from the “single center” to the “multi-center” in general. The number of hotels in the urban center area maintained rapid growth, and the density continued to improve, and the spatial agglomeration effect continued to strengthen. At the same time, Tongzhou, Shidu Scenic Area, Gubei Water Town, Miyun Reservoir and other areas have gradually formed the secondary agglomeration core of the hotel industry. The hotels in Beijing gradually developed from “the centripetal agglomeration” to “aggregation + diffusion”, and various hotels signifies two features of urban orientation and scenic orientation.

The natural geographical environment has shaped the overall layout of the hotel’s spatial distribution in Beijing. The number of hotels in the western and northern mountainous areas is small and the density is low, but that number in the eastern and southern plains is large and the density is high. And the hotel in the city distributes along the southwest-northeast direction. Furthermore, the socio-economic factors have an important impact on the hotel’s micro-location selection. The urban center area with frequent business activities, perfect public service facilities, complete tourism situations and sound traffic conditions is an ideal place for the layout of the hotel industry. However, when it has one of the above location advantages, it may also become a key choice for the space layout of the hotel industry.

Different factors have significant differences in the interpretation of hotel location selection, and the administrative agencies significantly influence the spatial layout of different types of hotels. The main transportation line and the subway line are the key areas of the hotel’s spatial layout, while the companies, bank insurance institutions, leisure and recreational facilities and the hotel industry show a significant spatial agglomeration distribution. In addition, social and economic factors, such as the land price and permanent population density, have a weak influence on the spatial distribution of the hotel industry; universities, train passenger capacity and other spatial distribution have no significant impact on the spatial distribution of hotels.

There are still certain shortcomings in this study. Firstly, the research scale focuses on the “district”, and the explanatory power of hotel micro-location selection needs to be improved. Second, limited by data sources, there are still some deficiency in the selection of influencing factors, especial for some factors that are difficult to quantify. In the future, we will better the data capture procedures, use different research methods to enhance the research results.

## Supporting information

S1 Table Spatiotemporal distribution data of Beijing's hotel industry (2% of all, N = 211). (PDF)

S2 Table The spatial distribution influence factor data in Beijing hotel industry (N = 16). (PDF)
