# Supplementary material for: The spatiotemporal evolution and influencing factors of hotel industry in the metropolitan area: An empirical study based on China
Source: PLoS One. 2020 May 19;15(5):e0231438. doi: 10.1371/journal.pone.0231438 (PMC7237036; doi:10.1371/journal.pone.0231438)
Supplement: S1 Table — (PDF) [file pone.0231438.s001.pdf]

S1 Table. Spatiotemporal distribution data of Beijing's hotel industry (2% of all, N = 211). This study included 10,543 hotels' opening time, lowest price (RMB) and location in Beijing, we used the stratified sampling method to select 2% of the data as supporting information and present it here (Table S1). All the data for these hotels comes from Elong.com and Qunar.com, which were compiled by the authors themselves. In this paper, the data sources of Fig. 1(a), Fig. 2, Fig. 3, Fig. 4, and Table 1, Table 2 are all from Table S1.

Serial number	Hotel code	The lowest price (RMB)	Opening time (Year)	Longitude (E°)	Latitude (N°)
1	X1	9897	2018	116.2561456	40.28135572
2	X51	2322	2018	116.3134107	40.6992545
3	X101	1665	2018	116.3654299	39.91241969
4	X151	1176	2018	116.4021807	39.85615002
5	X201	890	2018	116.1561049	39.75306854
6	X251	735	2018	116.3481012	39.85484141
7	X301	597	2018	116.2454396	40.22185941
8	X351	536	2018	116.4837291	39.862995
9	X401	493	2018	116.22706	40.270153
10	X451	453	2018	117.055236	40.492129
11	X501	404	2018	116.3881839	39.9384882
12	X551	378	2018	116.4019026	39.94245175
13	X601	354	2018	116.4236763	39.90327882
14	X651	325	2018	116.447149	39.956523
15	X701	298	2018	116.6724469	39.89973337
16	X751	280	2018	116.243192	40.080994
17	X801	265	2018	116.417165	39.939378
18	X851	250	2018	116.285461	39.82601435
19	X901	230	2018	116.2213925	40.21430962
20	X951	217	2018	116.2960556	39.97714187
21	X1001	203	2018	116.456247	39.86706782
22	X1051	196	2018	116.380662	39.948261
23	X1101	185	2018	117.2432576	40.65268233
24	X1151	175	2018	116.3522331	39.95513309
25	X1201	167	2018	116.5330981	40.42978853
26	X1251	156	2018	116.5239349	40.54533031
27	X1301	148	2018	116.3815561	40.00011231
28	X1351	136	2018	115.4965173	40.0276442
29	X1401	109	2018	116.3780101	39.94872149
30	X1451	81	2018	115.5155091	39.65370257
31	X1501	5153	2017	115.9337316	40.51747502
32	X1551	1665	2017	116.5238564	40.41720022
33	X1601	886	2017	116.6101442	40.05389563
34	X1651	692	2017	116.2739211	39.89881976
35	X1701	554	2017	116.1180567	40.46178498
36	X1751	498	2017	116.4163642	39.89576054
37	X1801	432	2017	117.2716133	40.65372135

38	X1851	385	2017	117.2500119	40.61689611
39	X1901	348	2017	116.3290415	39.95261228
40	X1951	320	2017	116.4399255	39.84468442
41	X2001	297	2017	117.2409044	40.65515049
42	X2051	276	2017	116.3292539	39.97297899
43	X2101	255	2017	116.511678	39.922981
44	X2151	224	2017	115.991665	40.36239564
45	X2201	199	2017	116.4425598	39.87447361
46	X2251	178	2017	117.208444	40.65247286
47	X2301	156	2017	116.1872872	40.34685323
48	X2351	140	2017	116.3876429	39.9453532
49	X2401	134	2017	116.2968708	39.99647523
50	X2451	127	2017	116.6089481	40.15806678
51	X2501	118	2017	116.4113793	39.94399325
52	X2551	111	2017	116.4405886	39.9178431
53	X2601	79	2017	116.593993	40.065242
54	X2651	1863	2016	117.251287	40.663784
55	X2701	1070	2016	116.0338956	39.92831635
56	X2751	690	2016	116.4068239	39.84495111
57	X2801	540	2016	116.2388722	39.58286288
58	X2851	446	2016	116.397141	39.90144
59	X2901	378	2016	117.2505619	40.31264957
60	X2951	340	2016	116.4334355	39.84556342
61	X3001	303	2016	116.4722571	39.88785689
62	X3051	275	2016	116.3155755	39.77234609
63	X3101	248	2016	116.6864566	40.3879503
64	X3151	223	2016	116.9794542	40.25648676
65	X3201	198	2016	115.7287744	40.00061227
66	X3251	178	2016	116.0252888	39.96378875
67	X3301	158	2016	116.1868951	39.79540196
68	X3351	144	2016	115.8945806	40.52737417
69	X3401	126	2016	117.4827537	40.64963762
70	X3451	108	2016	116.3563255	39.84321675
71	X3501	99	2016	116.3367791	39.973214
72	X3551	98	2016	115.4901273	39.65212564
73	X3601	90	2016	116.6881356	40.3878653
74	X3651	1672	2015	116.5189989	39.88297968
75	X3701	690	2015	116.624542	40.31811072
76	X3751	531	2015	116.3629785	39.95617416
77	X3801	460	2015	116.3881005	39.78271487
78	X3851	395	2015	116.3452071	39.87405445
79	X3901	358	2015	116.3151981	40.03423686
80	X3951	321	2015	116.3060838	39.88143001
81	X4001	289	2015	116.6285076	40.13237454

82	X4051	266	2015	116.2229963	39.89377823
83	X4101	243	2015	116.3509578	40.08505032
84	X4151	222	2015	116.6091451	40.0263652
85	X4201	205	2015	116.4125006	39.79615728
86	X4251	187	2015	116.4390977	39.92012405
87	X4301	167	2015	116.285712	39.876412
88	X4351	149	2015	116.5878912	39.94382936
89	X4401	129	2015	116.5215813	39.8435931
90	X4451	106	2015	116.3962528	40.17486915
91	X4501	89	2015	116.1617726	39.80051125
92	X4551	87	2015	117.2550228	40.6435134
93	X4601	67	2015	116.2276612	40.20667126
94	X4651	4955	2014	116.3828294	39.93382685
95	X4701	2953	2014	116.3875387	39.90079395
96	X4751	1002	2014	116.6095521	40.0251962
97	X4801	608	2014	116.8101959	40.52216864
98	X4851	446	2014	116.4003341	39.93364339
99	X4901	384	2014	116.6554378	39.86414054
100	X4951	347	2014	116.4456018	39.87429161
101	X5001	313	2014	116.7948251	40.60386678
102	X5051	288	2014	116.4843486	39.80020681
103	X5101	271	2014	116.587368	40.448863
104	X5151	254	2014	117.2418751	40.65821686
105	X5201	236	2014	116.5420402	39.90636485
106	X5251	208	2014	117.2259476	40.6578344
107	X5301	189	2014	115.543731	39.64974303
108	X5351	176	2014	117.2716133	40.65372135
109	X5401	158	2014	116.4599529	39.9581655
110	X5451	148	2014	116.4630339	39.98949198
111	X5501	136	2014	117.2101492	40.32883882
112	X5551	121	2014	116.7040004	40.5819707
113	X5601	101	2014	116.2680915	39.86304283
114	X5651	66	2014	116.6391376	40.13646263
115	X5701	2069	2013	116.6623614	40.39202584
116	X5751	892	2013	116.432268	39.94981573
117	X5801	476	2013	115.652374	40.003788
118	X5851	368	2013	116.33983	40.046982
119	X5901	313	2013	116.4831403	40.95823731
120	X5951	282	2013	116.4173419	40.55457381
121	X6001	258	2013	116.368607	39.899947
122	X6051	236	2013	116.5070539	39.91400231
123	X6101	207	2013	115.7320244	40.00283127
124	X6151	188	2013	116.4612518	39.87897017
125	X6201	169	2013	116.652769	40.3615642

126	X6251	152	2013	116.6974813	40.65565395
127	X6301	136	2013	116.4036233	39.93902225
128	X6351	117	2013	116.6560119	39.90593789
129	X6401	56	2013	116.0176166	40.53911937
130	X6451	1583	2012	116.4327286	39.9160511
131	X6501	808	2012	116.371835	39.92599132
132	X6551	469	2012	116.5591422	40.43054728
133	X6601	360	2012	116.1839329	40.04348099
134	X6651	309	2012	116.4690744	39.90159986
135	X6701	277	2012	116.4382678	40.11703824
136	X6751	245	2012	116.2770698	39.89434973
137	X6801	211	2012	117.239142	40.665766
138	X6851	185	2012	116.3971854	39.67879298
139	X6901	157	2012	116.5900324	40.36245379
140	X6951	136	2012	116.1798963	39.81342369
141	X7001	106	2012	116.5937201	40.21102807
142	X7051	1375	2011	115.9938676	40.36241448
143	X7101	899	2011	116.3854638	40.70255991
144	X7151	443	2011	116.4640445	39.90930647
145	X7201	334	2011	116.4151329	40.55527681
146	X7251	284	2011	116.2332931	40.21888494
147	X7301	229	2011	116.3338503	39.98707525
148	X7351	178	2011	116.6167857	40.37056305
149	X7401	147	2011	116.4916252	39.78957844
150	X7451	94	2011	115.5410654	39.66296967
151	X7501	987	2010	116.4568338	39.88434517
152	X7551	792	2010	116.38465	40.007505
153	X7601	560	2010	116.435669	39.933361
154	X7651	417	2010	116.4134388	39.95629888
155	X7701	354	2010	116.3183661	39.94967522
156	X7751	335	2010	116.31191	39.82100118
157	X7801	326	2010	115.991089	40.36368464
158	X7851	312	2010	115.9765717	40.35141636
159	X7901	296	2010	116.812899	40.54014556
160	X7951	271	2010	116.5945696	40.41254928
161	X8001	244	2010	116.3718614	39.77596748
162	X8051	210	2010	116.2882972	39.87590859
163	X8101	170	2010	116.6473659	39.75076025
164	X8151	145	2010	115.8749327	40.37414335
165	X8201	109	2010	115.6151413	39.65449499
166	X8251	772	2009	116.3725281	39.86762185
167	X8301	558	2009	116.5926821	40.29915154
168	X8351	323	2009	117.1146776	40.14954792
169	X8401	271	2009	116.6594401	40.49521412

170	X8451	218	2009	117.3206062	40.1847136
171	X8501	154	2009	116.2400926	40.66108203
172	X8551	2378	2008	116.4530584	39.9060639
173	X8601	738	2008	116.3488715	39.90224397
174	X8651	674	2008	116.2801011	39.84738986
175	X8701	600	2008	116.1473257	39.8033572
176	X8751	415	2008	116.3987166	39.93824875
177	X8801	350	2008	116.4022509	40.0073259
178	X8851	279	2008	115.6030006	40.01355432
179	X8901	231	2008	116.3061617	40.08702818
180	X8951	185	2008	116.4161855	39.90414459
181	X9001	118	2008	115.9647112	40.36050984
182	X9051	705	2007	116.3033321	39.93781382
183	X9101	623	2007	116.412095	39.91017483
184	X9151	338	2007	116.4029838	39.95456345
185	X9201	235	2007	116.5485573	39.97513638
186	X9251	126	2007	115.5801327	39.63534826
187	X9301	616	2006	116.4732885	39.8913122
188	X9351	351	2006	116.2418452	40.20227967
189	X9401	252	2006	116.6982166	40.44750059
190	X9451	128	2006	116.336597	39.997948
191	X9501	598	2005	116.0474493	39.97539068
192	X9551	354	2005	116.4113879	39.92842688
193	X9601	244	2005	116.6366712	40.73044064
194	X9651	136	2005	116.2223226	40.21417141
195	X9701	508	2004	116.3806621	39.86894385
196	X9751	203	2004	116.4537778	39.89533215
197	X9801	592	2003	116.3738771	39.95260549
198	X9851	163	2003	116.1544424	39.81653908
199	X9901	356	2002	116.35922	39.977231
200	X9951	582	2001	117.3801353	40.64018453
201	X10001	181	2001	116.4610604	40.41364445
202	X10051	431	2000	116.2983607	39.9444659
203	X10101	90	2000	115.6002655	39.64057636
204	X10151	776	1998	116.4534778	39.86857563
205	X10201	1662	1997	116.3799574	39.93218798
206	X10251	180	1997	117.2411421	40.65729686
207	X10301	499	1995	116.41116	39.91522183
208	X10351	562	1993	116.383474	39.924057
209	X10401	1399	1990	116.4529502	39.90889479
210	X10451	199	1988	116.4437356	39.88289717
211	X10501	552	1983	116.4255172	39.91044547
