# Supplementary material for: The spatiotemporal evolution and influencing factors of hotel industry in the metropolitan area: An empirical study based on China
Source: PLoS One. 2020 May 19;15(5):e0231438. doi: 10.1371/journal.pone.0231438 (PMC7237036; doi:10.1371/journal.pone.0231438)
Supplement: S2 Table — (PDF) [file pone.0231438.s002.pdf]

S2 Table. The spatial distribution influence factor data in Beijing hotel industry (N=16). This table is specific data on the influence factors of spatial distribution of the hotel industry in Beijing, include regional economy, traffic condition, business condition, public service, and tourism situation. The research data sources are: ① *Beijing Regional Statistical Yearbook* (2018), ② government department public data collation, ③ Baidu POI data, and ④ train passenger capacity and tourism resource endowment reference related research in accordance with the assignment method. We have already described it in detail in our paper, so we will not cover it here. In this paper, the data of Table 4 and Fig. 5 are from Table S2.

Index	Factor	Dongcheng	Xicheng	Chaoyang	Haidian	Fengtai	Shijingshan	Shunyi	Tongzhou
Regional economy	X1: Per capita GDP (ten thousand yuan)	16.59	6.09	29.00	17.10	7.47	7.20	35.38	6.41
	X2: Per capita disposable income (ten thousand yuan)	7.07	3.50	7.55	7.39	3.68	6.01	8.17	3.94
	X3: Permanent population density (person/km2)	7654	222	19637	8341	195	6890	23300	597
	X4: Land price (ten thousand yuan/m2)	5.58	2.64	13.11	6.99	3.58	7.01	13.11	2.84
Traffic condition	X5: Train passenger capacity (train number)	26	14	954	0	38	6824	0	40
	X6: Subway station (quantity)	97	0	37	60	0	42	36	10
	X7: Per capita car ownership (car/person)	0.61	1.21	3.06	0.72	0.86	0.77	1.64	0.75
	X8: Road density (km / km2)	4.25	0.96	10.04	3.94	0.79	3.62	7.58	1.56
Business condition	X9: Company (quantity)	16462	1075	2956	7203	1145	3938	2652	1593
	X10: Restaurant (quantity)	13821	1069	4336	7903	1067	5580	4286	1839
	X11: Leisure and recreational facilities (quantity)	3458	1367	1135	2741	1589	1806	1396	1046
	X12: Convenient service facilities (quantity)	10565	951	2887	5960	722	5375	2801	1756
Public service	X13: Administrative agency (quantity)	2434	692	1544	2305	620	1529	1534	913
	X14: Bank and Insurance (quantity)	1006	122	378	821	115	366	534	176
	X15: Hospital (quantity)	165	13	65	91	12	74	50	34
	X16: University and college (quantity)	16	0	3	34	1	6	6	7
Tourism situation	X17: Tourism resource (score)	178	126	40	140	126	78	160	150
	X18: Travel agency (quantity)	1176	32	384	241	22	362	240	58
	X19: Inbound tourists (ten thousand persons)	180.70	0.23	110.51	35.05	0.78	6.55	21.69	0.04

Index	Factor	Daxing	Fangshan	Mentougou	Changping	Pinggu	Miyun	Huairou	Yanqing
Regional economy	X1: Per capita GDP (ten thousand yuan)	4.36	4.33	4.30	5.28	15.20	5.28	5.42	9.90
	X2: Per capita disposable income (ten thousand yuan)	3.39	4.53	4.35	4.55	3.36	3.60	4.98	7.12
	X3: Permanent population density (person/km2)	175	1568	1572	1741	1156	481	222	6881
	X4: Land price (ten thousand yuan/m2)	1.16	4.30	5.24	2.72	2.25	1.35	3.43	4.47
Traffic condition	X5: Train passenger capacity (train number)	0	16	26	22	10	0	0	8
	X6: Subway station (quantity)	0	20	16	18	8	0	0	9
	X7: Per capita car ownership (car/person)	1.49	0.39	0.42	0.48	0.55	1.88	1.32	0.88
	X8: Road density (km / km2)	0.92	1.61	2.77	2.63	2.90	1.75	0.49	3.20
Business condition	X9: Company (quantity)	393	2278	4223	3885	2916	815	278	721
	X10: Restaurant (quantity)	634	3893	3158	2912	2265	607	393	1098
	X11: Leisure and recreational facilities (quantity)	659	1562	1074	755	711	592	472	505
	X12: Convenient service facilities (quantity)	488	3531	2525	2675	1730	653	352	1206
Public service	X13: Administrative agency (quantity)	518	912	1207	983	1060	546	406	535
	X14: Bank and Insurance (quantity)	72	224	260	219	211	118	66	125
	X15: Hospital (quantity)	4	79	42	24	15	7	13	25
	X16: University and college (quantity)	1	17	11	5	2	0	0	4
Tourism situation	X17: Tourism resource (score)	94	120	32	28	50	58	108	22
	X18: Travel agency (quantity)	18	105	53	103	53	20	24	41
	X19: Inbound tourists (ten thousand persons)	0.02	2.32	10.49	0.69	19.68	0.02	0.06	3.68
